# Sleep Difficulties Among COVID-19 Frontline Healthcare Workers

**DOI:** 10.3389/fpsyt.2022.838825

**Published:** 2022-04-29

**Authors:** Rony Cleper, Nimrod Hertz-Palmor, Mariela Mosheva, Ilanit Hasson-Ohayon, Rachel Kaplan, Yitshak Kreiss, Arnon Afek, Itai M. Pessach, Doron Gothelf, Raz Gross

**Affiliations:** ^1^Sackler Faculty of Medicine, Tel Aviv University, Tel Aviv, Israel; ^2^The Chaim Sheba Medical Center, Ramat Gan, Israel; ^3^School of Psychological Sciences, Tel Aviv University, Tel Aviv, Israel; ^4^Department of Psychology, Bar-Ilan University, Ramat Gan, Israel

**Keywords:** sleep, sleep difficulties, COVID-19, health care workers (HCW), COVID-19 outbreak, sleep disorders, health care staff, stress

## Abstract

**Objective:**

To identify COVID-19 work-related stressors and experiences associated with sleep difficulties in HCW, and to assess the role of depression and traumatic stress in this association.

**Methods:**

A cross-sectional study of HCW using self-report questionnaires, during the first peak of the pandemic in Israel (April 2020), conducted in a large tertiary medical center in Israel. Study population included 189 physicians and nurses working in designated COVID-19 wards and a comparison group of 643 HCW. Mean age of the total sample was 41.7 ± 11.1, 67% were female, 42.1% physicians, with overall mean number of years of professional experience 14.2 ± 20. The exposure was working in COVID-19 wards and related specific stressors and negative experiences. Primary outcome measurement was the Insomnia Severity Index (ISI). Secondary outcomes included the Primary Care-Post Traumatic Stress Disorder Screen (PC-PTSD-5); the Patient Health Questionnaire-9 (PHQ-9) for depression; the anxiety module of the Patient-Reported Outcomes Measurement Information System (PROMIS); Pandemic-Related Stress Factors (PRSF) and witnessing patient suffering and death.

**Results:**

Compared with non-COVID-19 HCW, COVID-19 HCW were more likely to be male (41.3% vs. 30.7%) and younger (36.91 ± 8.81 vs. 43.14 ± 11.35 years). COVID-19 HCW reported higher prevalence of sleep difficulties: 63% vs. 50.7% in the non-COVID group (OR 1.62, 95% CI 1.15–2.29, *p* = 0.006), mostly difficulty maintaining sleep: 26.5% vs. 18.5% (OR 1.65, 95% CI 1.11–2.44, *p* = 0.012). Negative COVID-19 work-related experiences, specifically witnessing patient physical suffering and death, partially explained the association. Although past psychological problems and current depression and PTSD were associated with difficulty maintaining sleep, the main association remained robust also after controlling for those conditions in the full model.

**Conclusion and Relevance:**

COVID-19 frontline HCW were more likely to report sleep difficulties, mainly difficulty maintaining sleep, as compared with non-COVID-19 HCW working at the same hospital. Negative patient-care related experiences likely mediated the increased probability for those difficulties. Future research is needed to elucidate the long-term trajectories of sleep difficulties among HCW during large scale outbreaks, and to identify risk factors for their persistence.

## Introduction

The COVID-19 pandemic continues to challenge health care workers, as a new COVID variants keep emerging. Working at the frontline of this global pandemic is highly stressful and can significantly impact various aspects of daily life, including sleep quality and quantity (1). Recently, studies from several countries have shown high prevalence of insomnia and of sleep difficulties among hospital medical staff involved in the pandemic, especially among female staff and those with psychological symptoms and with lower educational attainment (2–6).

The association between stressful workplace experiences and poor sleep quality, independent of home-related stress, was previously reported (7). Sleep difficulties were found to be associated with poorer cognitive performance (8, 9), increased numbers of occupational accidents and injuries (10, 11) and car crashes (12, 13). Impaired cognitive and motor skills were found among hospital residents, even with acute, short-standing sleep loss (14). Sleep deprivation was found to negatively impacts surgeons’ technical skills, with obvious implications for patient safety (15), and in the longer term, it was associated with neurological dysfunction and even death (16). In addition, sleep deprivation was found to predict a wide variety of mental disorders (17), including increased risk for stress (18), depression (19), suicidality (20, 21), and also psychotic experiences (22). Furthermore, during stressful events, short sleep was associated with diminished coping with stress (23), increased susceptibility for posttraumatic stress disorder (PTSD) (24–27), as well as for anxiety and hypomania (28).

Although previous research on frontline COVID-19 health care workers (HCW) found high prevalence of sleep difficulties, detailed data on the nature of those difficulties and on associated risk factors are still limited. Furthermore, it is unclear whether sleep difficulties are an independent outcome among COVID-19 HCW or merely a manifestation of other mental health outcomes, such as depression and PTSD. We set to compare the frequency of sleep difficulties among COVID-19 and non-COVID-19 HCW during the first peak of the pandemic in Israel, to identify factors associated with those difficulties, and to investigate whether they occur independent of PTSD and depression. We aim to explore the association between factors related to work at COVID-19 wards and reported sleep difficulties. More specifically, we ask whether negative experiences that are more prevalent among COVID-19 HCW play a role as intermediates in that association.

## Materials and Methods

We conducted a single-center, cross-sectional study among physicians and nurses working at the Sheba Medical Center, a large tertiary medical center in central Israel. The study was conducted between April 19–23, 2020. During this period the total number of confirmed COVID-19 cases in Israel peaked from 13,319 (April 19) to 14,511 (April 23).

This study followed the standards and ethics of the American Association for Public Opinion Research reporting guidelines (29) and the Strengthening the Reporting of Observational Studies in Epidemiology (STROBE) reporting guidelines (30). The protocol was approved by the Institutional Review Board of the Sheba Medical Center. Participation in the study was solely voluntary. All participants signed an electronic consent form. The data collected did not include personal identifiers (e.g., name, home address, phone number or email).

### Participants

As part of the preparation for the surge in COVID-19 confirmed cases in Israel, specialized COVID-19 care wards were set up and isolated from other care areas in the hospital. Designated teams were allocated for COVID-19 containment wards, as well as two intensive care units, a designated emergency department, five inpatient wards and a psychiatry ward were assembled for the expected COVID-19 patients, totaling almost 400 specialized beds. We aimed at oversampling HCW working in COVID-19 wards (31). A total of 189 HCW from designated COVID-19 departments and 643 non-designated COVID-19 ward HCW (comparison group) responded to the survey, a total of 828 HCW. Sample flow across COVID-19 and non-COVID-19 teams is presented in Supplementary Figure 1.

### Study Measures

The participants completed a self-administered anonymous questionnaire digitally through a secured digital platform (Qualtrics). The questionnaire included information on current ward (COVID-19 containment wards or regular wards), sociodemographic characteristics, general and mental health items, and a question about having to go into quarantine (yes/no).

Sleep difficulties were measured with the validated Hebrew version of the Insomnia Severity Index (ISI) (32, 33). Response options of the ISI questions were collapsed into dichotomous values (yes/no) for each of the three ISI items.

**FIGURE 1:**
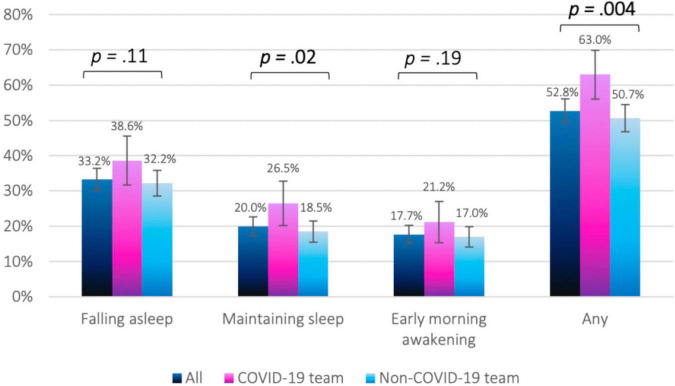
Distribution of sleep difficulties by study group.

The prevalence of having at least one form of sleep difficulty was significantly higher among COVID-19 vs. non-COVID-19 HCW (63% and 50.7%, respectively, *p* = 0.004). Specifically, COVID-19 HCW were more likely to experience difficulty maintaining sleep (26.5% and 18.5%, respectively, *p* = 0.02) as presented in Figure 1.

Traumatic stress during the past month was assessed with the validated Hebrew version of the Primary Care PTSD Screen for DSM-5 (PC-PTSD-5) (34). The PC-PTSD-5 has a score range of 0-5, and we set a cutoff of 3 to define traumatic stress symptoms (35). Participants were asked to respond about their traumatic symptoms specifically in relation to the COVID-19 pandemic.

Depression was assessed with the Hebrew version of the well-validated Patient Health Questionnaire-9 (PHQ-9) (36). The PHQ-9 has a score range of 0–27, and a score of ≥10 was used to identify probable depression in the current study (37).

Anxiety among HCW was assessed by means of the 8-item Hebrew version of the National institution of Mental Health (NIMH) Patient-Reported Outcomes Measurement Information System (PROMIS) anxiety module (38–41). PROMIS has an established coding system validated by the NIMH, with standardized “T” scores ranging between 36.3–82.7 (“PROMIS^®^ Scoring Manuals.,” n.d.). The cutoff point for probable anxiety was set at T ≥62.3, considered as equivalent to the GAD-7 standard cutoff score for moderate anxiety (=10) (40).

Pandemic-related stress factors (PRSF) were measured with an inventory compiled from questions which were proved to be pertinent in research carried out during the SARS and N1H1 pandemics (42–44). A 4-point Likert-type scale was used for scoring the items (from 0 = never to 3 = always). Questions concerning negative experiences included exposure to patients’ physical and mental suffering, the number of patient deaths witnessed over the past month (none, one, more than one) (45), and the self-perceived physical health question from the 12-item Medical Outcomes Study (MOS) Short-Form Health Status Survey (SF-12) (Hebrew version) (poor, fair, good, very good, excellent) (46).

### Statistical Analysis

Descriptive statistics were used to describe the sample. We used chi-square tests to compare sociodemographic characteristics, sleep difficulties, prevalence of specific traumatic stress symptoms, probable depression (PHQ-9 score ≥10), probable PTSD (PC-PTSD-5 score ≥3) and probable anxiety (PROMIS score ≥62.3) between COVID-19 and non-COVID-19 HCW. *T*-tests were used to compare PHQ-9 and PROMIS anxiety module mean scores between study teams. We recoded ordinal variables with multiple categories (e.g., PRSF items, exposure to patient suffering) as categorical, with 1 and 0 representing high and low categories, respectively. Missing values in the PROMIS Anxiety module and the PHQ-9 questionnaire were imputed by the group mean score (47). Logistic regression was used to compare the likelihood of sleep difficulties among COVID-19 and non-COVID-19 wards. Multiple logistic models were used to assess potential mediators and confounders. Adjusted ORs and 95% *CIs* were computed, with non-COVID-19 team as the reference group.

Next, we conducted a three-step hierarchical logistic regression, to explore the role of potential confounders and mediators in the association between working at COVID-19 wards and sleep difficulties. Covariates were included based on the following criteria: (1) basic sociodemographic characteristics (age, sex, profession and professional experience); (2) theoretical and empirical framework developed based on research carried out during previous and current pandemics (witnessing patient suffering and death, and past psychological problems); and (3) variables that did not have considerable effect on the association between study group and sleep difficulties (PRSF and financial concerns) were not included in the hierarchical model. As model fitting *via* logistic regression is sensitive to collinearities among independent variables, we decontaminated the strongly correlated witnessing patient physical suffering and witnessing patient death variables from common variance by regressing them out of each other and including their standardized residuals in the final model (48). This method does not affect model’s predictability, and therefore R2 remains unchanged compared with the non-residualized model but reduces multicollinearity and extracts the unique variance explained by each predictor, resulting in purified more powerful coefficients.

A three-step hierarchical logistic regression was used to test whether probable depression and PTSD, either alone or combined, accounted for the increased likelihood of sleep difficulties among COVID-19 HCW. Alpha was set at 0.05, and all tests were 2-tailed. Statistical analyses were conducted with IBM SPSS V25 software.

## Results

The analytic sample included a total of 828 HCW (42.1% physician, 57.9% nurses), of whom 189 worked in the COVID-19 wards (42.3% of total COVID-19 team members in the hospital) and 639 in the non-COVID-19 wards (20.1% of total non-COVID-19 teams). Detailed description of the study sample are reported elsewhere (49). The main characteristics and work-related experiences of the study groups are presented in Table 1.

**TABLE 1:** Sociodemographic and clinical characteristics of the sample.

Characteristic	Total sample (*n* = 828)	COVID-19 team (*n* = 189)	non-COVID-19 team (*n* = 639)	*P*-value†
Age, Mean (SD), y	41.7 (11.1)	36.9 (8.8)	43.1 (11.3)	<0.0001
	n(%)	n (%)	n (%)	
Sex, female	557 (67.0%)	111 (58.7%)	443 (69.3%)	0.006
Physician/nurse	349/479 (42.1/57.9)	73/116 (38.6/61.4)	276/363 (43.2/56.8)	0.264
Quarantined	139 (16.7)	41 (21.7)	78 (15.3)	0.038
Having medical conditions	225 (26.7)	47 (25.0)	178 (27.9)	0.439
Past psychological problems	297 (35.2)	82 (43.6)	215 (33.6)	0.012
Marital status				<0.0001
Single	157 (18.9)	59 (31.2)	98 (15.3)	
Married	584 (70.6)	114 (60.3)	470 (73.5)	
Divorced	61 (7.3)	11 (5.8)	50 (7.8)	
Other	26 (3.1)	5 (2.6)	21 (3.2)	
Living alone	122 (14.8)	44 (23.7)	78 (12.8)	<0.0001
Religion				<0.0001
Jewish	687 (82.7)	131 (69.7)	556 (86.5)	
Muslim	69 (8.3)	36 (19.1)	33 (5.1)	
Christian	6 (0.7)	2 (1.1)	4 (0.6)	
Atheist	56 (6.7)	17 (9.0)	39 (6.1)	
Other	13 (1.6)	2 (1.1)	11 (1.7)	
Professional experience, mean (SD), years	14.2 (20)	9.9 (9.4)	15.4 (12.3)	<0.0001
ISI items				
- Any sleep difficulties	445 (52.8)	119 (63.0)	326 (50.7)	0.004
-Difficulties falling asleep	280 (33.2)	73 (38.6)	207 (32.2)	0.11
-Maintaining sleep	169 (20.0)	50 (26.5)	119 (18.5)	0.02
-Early morning awakening	149 (17.7)	40 (21.2)	109 (17.0)	0.19
PC-PTSD-5, No. of symptoms, median (IQR)		1 (0–2)	0 (0–1)	
-0 symptoms	459 (55.4)	88 (46.6)	371 (57.7)	0.004
-1–2 symptoms	253 (30.5)	67 (35.5)	186 (28.9)	
-3–5 symptoms	115 (13.8)	32 (16.9)	83 (12.9)	
PHQ-9, mean (SD)		6.6 (4.9)	5.4 (5.0)	0.079
-PHQ-9 ≥10	169 (20.4)	47 (25.0)	113 (17.7)	0.025
PROMIS Anxiety, mean (SD)		58.2 (7.8)	57.9 (7.8)	0.427
-PROMIS ≥62.3	277 (33.4)	70 (37.0)	207 (32.2)	0.234
Pandemic-related stress factors (endorsing “often” or “always”)				
Anxiety about being infected	165 (19.9)	34 (18.1)	131 (20.4)	0.350
Anxiety about infecting family	403 (48.6)	102 (53.9)	301 (47.1)	0.014
Lack of knowledge about infectiveness and virulence	149 (17.3)	37 (19.6)	112 (17.5)	0.700
Lack of knowledge about prevention and protection	120 (14.4)	28 (14.8)	92 (14.4)	0.126
Financial concerns	267 (32.2)	59 (31.7)	208 (32.6)	0.387
Negative experiences				
High exposure to physical suffering of patients (often\always)	637 (76.9)	154 (81.5)	483 (75.6)	0.004
High exposure to mental suffering of patients (often\always)	650 (78.5)	140 (74.9)	510 (80.0)	0.442
Negative self-perceived health (fair\poor)	79 (9.5)	17 (9.0)	62 (9.8)	0.191
Witnessing patient death	274 (33.0)	95 (50.2)	179 (24.7)	<0.001
-None	572 (69.0)	92 (48.7)	480 (74.7)	<0.001
-1	123 (14.8)	34 (18.0)	89 (13.8)	
-≥2	131 (15.8)	61 (32.2)	70 (10.9)	

*COVID-19, coronavirus disease 2019; ISI, Insomnia Severity Index; PC-PTSD-5, Primary Care-Post Traumatic Stress Disorder Screen for DSM-5; PHQ-9, Patient Health Questionnaire-9; PROMIS, the anxiety module of the Patient-Reported Outcomes Measurement Information System.*

*†PC-PTSD-5, Pandemic-related stress factors and negative experiences were tested with Mann–Whitney U test for independent samples. PHQ-9 and PROMIS Anxiety were tested with ANCOVA adjusted for age, sex and physician\nurse. Above\below cutoff proportions of PHQ-9 and PROMIS Anxiety were tested with Z-test for 2-populations proportions.*

COVID-19 HCW were more likely to experience any sleep difficulties (OR 1.62, 95% CI 1.15–2.29, *p* = 0.006). Difficulty maintaining sleep emerged as the strongest and most significant finding (OR 1.65, 95% CI 1.11–2.44, *p* = 0.012). These associations persisted in the multivariate models that adjusted for age, sex, and profession (model 1), and for PRSF items (anxiety about being infected, anxiety about infecting family, lack of knowledge about infectiveness and virulence, and lack of knowledge about prevention and protection) (model 2). The effect of financial concerns on the associations was negligible. The association between working in COVID-19 wards and difficulty maintaining sleep was attenuated when negative experiences were added to the model as shown in Table 2.

**TABLE 2:** Crude and adjusted odds ratios for sleep difficulties among COVID-19 vs. non- COVID-19 teams.

		Difficulty falling asleep		Difficulty maintaining sleep		Early morning awakening		Any sleep difficulties	
		COVID 19 team	Non-COVID-19 team	COVID 19 team	Non- COVID-19 team	COVID 19 team	Non- COVID-19 team	COVID 19 team	Non- COVID-19 team
**Unadjusted**									
	OR (CI 95%)	1.35 (0.95–1.92)	1.00	1.65 (1.11–2.44)	1.00	1.28 (0.84–1.96)	1.00	1.62 (1.15–2.29)	1.00
	P	0.089		0.012		0.249		0.006	
**Adjusted models**									
Model 1	aOR (CI 95%)	1.17 (0.81–1.70)	1.00	1.56 (1.04–2.35)	1.00	1.20 (0.77–1.87)	1.00	1.52 (1.06–2.19)	1.00
	P	0.378		0.030		0.410		0.022	
Model 2	aOR (CI 95%)	1.16 (0.80–1.68)	1.00	1.59 (1.05–2.40)	1.00	1.25 (0.80–1.96)	1.00	1.56 (1.08–2.26)	1.00
	P	0.419		0.026		0.322		0.017	
Model 3	aOR (CI 95%)	1.21 (0.84–1.75)	1.00	1.59 (1.06–2.40)	1.00	1.21 (0.77–1.89)	1.00	1.58 (1.10–2.27)	1.00
	P	0.289		0.024		0.391		0.013	
Model 4	aOR (CI 95%)	1.17 (0.80–1.72)	1.00	1.46 (0.96–2.23)	1.00	1.16 (0.73–1.85)	1.00	1.54 (1.05–2.25)	1.00
	P	0.398		0.075		0.504		0.025	
Model 5	aOR (CI 95%)	1.15 (0.78–1.70)	1.00	1.49 (0.97–2.29)	1.00	1.21 (0.75–1.93)	1.00	1.56 (1.06–2.29)	1.00
	P	0.786		0.067		0.422		0.024	

*Model 1: Adjusted for age, sex, profession and years in profession.*

Model 2: Adjusted for age, sex, profession, years in profession and PRSF*.

*Model 3: Adjusted for age, sex, profession, years in profession and financial concerns.*

*Model 4: Adjusted for age, sex, profession, years in profession and negative experiences.*

Model 5: Adjusted for age, sex, profession, years in profession, PRSF*, financial concerns and negative experiences.

*PRSF items include: anxiety about being infected; anxiety about infecting family; lack of knowledge about infectiveness and virulence; lack of knowledge about prevention and protection.

*CI, confidence interval; OR, odds ratio; COVID-19, coronavirus disease 2019; aOR, adjusted odds ratio; PRSF, Pandemic-Related Stress Factors.*

Next, we focused on exploring the association between difficulty maintaining sleep and variables selected according to the aforementioned criteria, using three-step hierarchical logistic regression model, presented in Table 3. We found that the main effect of work in COVID-19 ward on difficulty maintaining sleep was considerably attenuated after adjusting for witnessing patient physical suffering and death were added to the model. Adding self-reported past psychological problems to the model, which was found to be associated with difficulty maintaining sleep, further attenuated the association in the full model (OR 1.35, 95% CI 0.89–2.06, *p* = 0.162).

**TABLE 3:** Factors associated with likelihood of difficulty maintaining sleep in COVID-19 and non-COVID-19 teams.

	Step I		Step II		Step III	
Variable	OR (95% CI)	*P*	OR (95% CI)	*P*	OR (95% CI)	*P*
COVID-19 team	1.59 (1.06, 2.38)	**0.024**	1.43 (0.94, 2.19)	0.093	1.35 (0.89, 2.06)	0.162
Age (older)	0.86 (0.56, 1.31)	0.477	0.91 (0.59, 1.41)	0.678	0.89 (0.58, 1.39)	0.627
Sex (female)	0.93 (0.62, 1.39)	0.711	0.98 (0.65, 1.48)	0.925	0.91 (0.60, 1.39)	0.678
Physician/nurse (physician)	0.67 (0.45, 1.00)	**0.050**	0.69 (0.46, 1.03)	0.066	0.66 (0.44, 0.99)	**0.049**
Professional experience (longer)	0.89 (0.58, 1.36)	0.578	0.95 (0.62, 1.46)	0.824	0.96 (0.62, 1.48)	0.841
Witnessing patient physical suffering	–		1.40 (1.15, 1.71)	**0.001**	1.37 (1.12, 1.67)	**0.002**
Witnessing patient death	–		1.24 (1.04, 1.48)	**0.017**	1.23 (1.03, 1.47)	**0.022**
Past psychological problems	–		–	–	1.68 (1.17, 2.43)	**0.005**

*Non-COVID-19 is the reference group (OR = 1.00).*

*Age and professional experience were computed as standardized residualized from each other, due to co-linearity.*

*CI, confidence interval; COVID-19, coronavirus disease 2019; OR, odds ratio.*

*The bold figures are highlighted for significant p-values (< 0.05).*

Lastly, although difficulty maintaining sleep was associated with both probable depression and PTSD (OR 3.36 and 3.12, respectively), those conditions did not account for the increased likelihood of difficulty maintaining sleep among COVID-19 HCW as shown in Table 4.

**TABLE 4:** The association between difficulty maintaining sleep and PTSD and depression in COVID-19 and non-COVID-19 healthcare workers.

Difficulty maintaining sleep								
	Step I		Step IIa depression		Step IIb PTSD		Step III depression and PTSD	
Variable	OR (95% CI)	*P*	OR (95% CI)	*P*	OR (95% CI)	*P*	OR (95% CI)	*P*
COVID-19 team	1.57 (1.05, 2.36)	**0.027**	1.53 (1.01, 2.32)	0.044	1.61 (1.07, 2.43)	0.023	1.57 (1.03, 2.39)	**0.035**
Age (older)	0.86 (0.56, 1.32)	0.496	1.09 (0.70, 1.71)	0.700	1.02 (0.66, 1.58)	0.928	1.18 (0.75, 1.85)	0.480
Sex (female)	0.93 (0.62, 1.39)	0.726	0.87 (0.57, 1.31)	0.502	0.88 (0.58, 1.33)	0.556	0.85 (0.56, 1.30)	0.457
Physician/nurse (physician)	0.66 (0.44, 0.98)	**0.042**	0.73 (0.48, 1.11)	0.137	0.71 (0.47, 1.07)	0.099	0.75 (0.49, 1.13)	0.169
Professional experience (longer)	0.89 (0.58, 1.37)	0.606	1.10 (0.70, 1.71)	0.684	1.05 (0.68, 1.62)	0.833	1.17 (0.75, 1.84)	0.480
Depression	–	–	3.36 (2.23, 5.04)	**<0.001**	–	–	2.68 (1.72, 4.19)	**<0.001**
PTSD	–	–	–	–	3.12 (1.98, 4.91)	**<0.001**	2.03 (1.23, 3.35)	**0.006**

*Non-COVID-19 is the reference group (OR = 1.00).*

*Age and professional experience were computed as standardized residualized from each other, due to collinearity.*

*CI, confidence interval; COVID-19, coronavirus disease 2019; OR, odds ratio.*

*The bold figures are highlighted for significant p-values (< 0.05).*

## Discussion

The main finding that emerged from our data was that COVID-19 HCW experienced higher prevalence of sleep difficulties, specifically difficulty maintaining sleep, compared with non-COVID-19 HCW in a large tertiary medical center in central Israel, during the first peak of COVID-19 pandemic. Although sleep difficulties among frontline HCW were previously reported in the current and previous pandemics (2, 3, 50–57), there are no published data on the role of mediating factors that might explain the association between work in COVID-19 wards and sleep difficulties.

We found that negative experiences, most notably witnessing physical suffering by the patient and patient death, accounted partially for the association between working in COVID-19 ward and difficulty maintaining sleep. The role of those two negative experiences as intermediates persisted also after adjusting for the potential confounding effect of past psychological problems. The mediation effect was unique for those negative experiences and was not found for other variables related to the pandemic, such as PRSF and financial concerns. It is plausible that witnessing patient suffering and death induced distressing dreams (nightmares), which interrupted REM sleep (58). Interestingly, the association between working in COVID-19 ward and difficulty maintaining sleep in our sample could not be attributed to current probable depression or PTSD, despite the fact that sleep difficulties are common symptoms in both disorders.

A plausible contributor to the increase in sleep difficulties among frontline HCW during the pandemic is workplace violence, shown in other studies to have increased during the pandemic (59–61).

The main strength of our study is that both study group and comparison group (COVID-19 team and non-COVID-19 team, respectably) were sampled from the same underlying cohort of physicians and nurses, sharing occupational, organizational and hospital leadership features. Additional strengths of our study include: (1) the ‘real-time’ nature of our data, as it were collected during the first peak of the pandemic in Israel, and not retrospectively, thus reducing the likelihood of recall bias; (2) availability of objective information on study group allocation; (3) study outcomes were measured by means of well-validated instruments; and (4) a very low proportion of missing data.

Our findings have several potential implications for the frontline workforce during a pandemic. First, screening for sleep difficulties among COVID-19 HCW, especially those exposed to negative experiences, could prompt targeted early intervention, especially in light of reported beneficial impact of fatigue training for improving personnel and patient safety, and reducing stress and burnout among HCW (62). Second, achieving trauma-induced sleep disorder normalization was shown to reduced risk of PTSD (63, 64), frequently reported among COVID-19 HCW (41, 65, 66). Additionally, integrating occupational mental health programs at healthcare settings, was shown to help alleviating pandemic-related sleep difficulties (67).

### Limitations

Our study has several limitations. First, conclusion about directionality is limited by the cross-sectional study design. However, it is unlikely that assignment of HCW to COVID-19 wards was conditioned on history of sleep difficulties. Second, the higher prevalence of sleep difficulties among the COVID-19 HCW might be partially explained by the higher workload in COVID-19 wards, especially considering the reduced work volume in the non-COVID-19 wards during that time. Third, the study was conducted in a single medical center in Israel during the first wave of the pandemic, and therefore the generalizability of our findings might be limited. Fourth, non-responders had slightly different sociodemographic characteristics. Fifth, data were collected by means of self-report questionnaires rather than clinical interviews.

## Conclusion

We found that COVID-19 frontline HCW were more likely to report sleep difficulties, mainly difficulty maintaining sleep, as compared with HCW working in regular wards at the same hospital, and that negative patient-care related experiences likely mediated the increased likelihood for those difficulties. Future research is needed to elucidate the long-term trajectories of sleep difficulties among HCW caring for COVID-19 patients, and to identify antecedents and risk factors for persistence of those difficulties.

## Data Availability Statement

The raw data supporting the conclusions of this article will be made available by the authors, without undue reservation.

## Author Contributions

RC, MM, RG, NH-P, DG, IP: conceiving and designing the study. MM, NH-P, and IP: data collection. NH-P: statistical analyses. RC, RG, NH-P, IH-O, RK, RC, YK, DG, and IP: data interpretation. RC and RG: writing the final manuscript. All authors contributed to the article and approved the submitted version.

## Conflict of Interest

The authors declare that the research was conducted in the absence of any commercial or financial relationships that could be construed as a potential conflict of interest.

## Publisher’s Note

All claims expressed in this article are solely those of the authors and do not necessarily represent those of their affiliated organizations, or those of the publisher, the editors and the reviewers. Any product that may be evaluated in this article, or claim that may be made by its manufacturer, is not guaranteed or endorsed by the publisher.

## Supplementary Material

Supplementary Figure 1 Flow diagram for study participants.
