# Supplementary figures and images for: Sleep Difficulties Among COVID-19 Frontline Healthcare Workers
Source: Front Psychiatry. 2022 Apr 29;13:838825. doi: 10.3389/fpsyt.2022.838825 (PMC9098971; doi:10.3389/fpsyt.2022.838825)

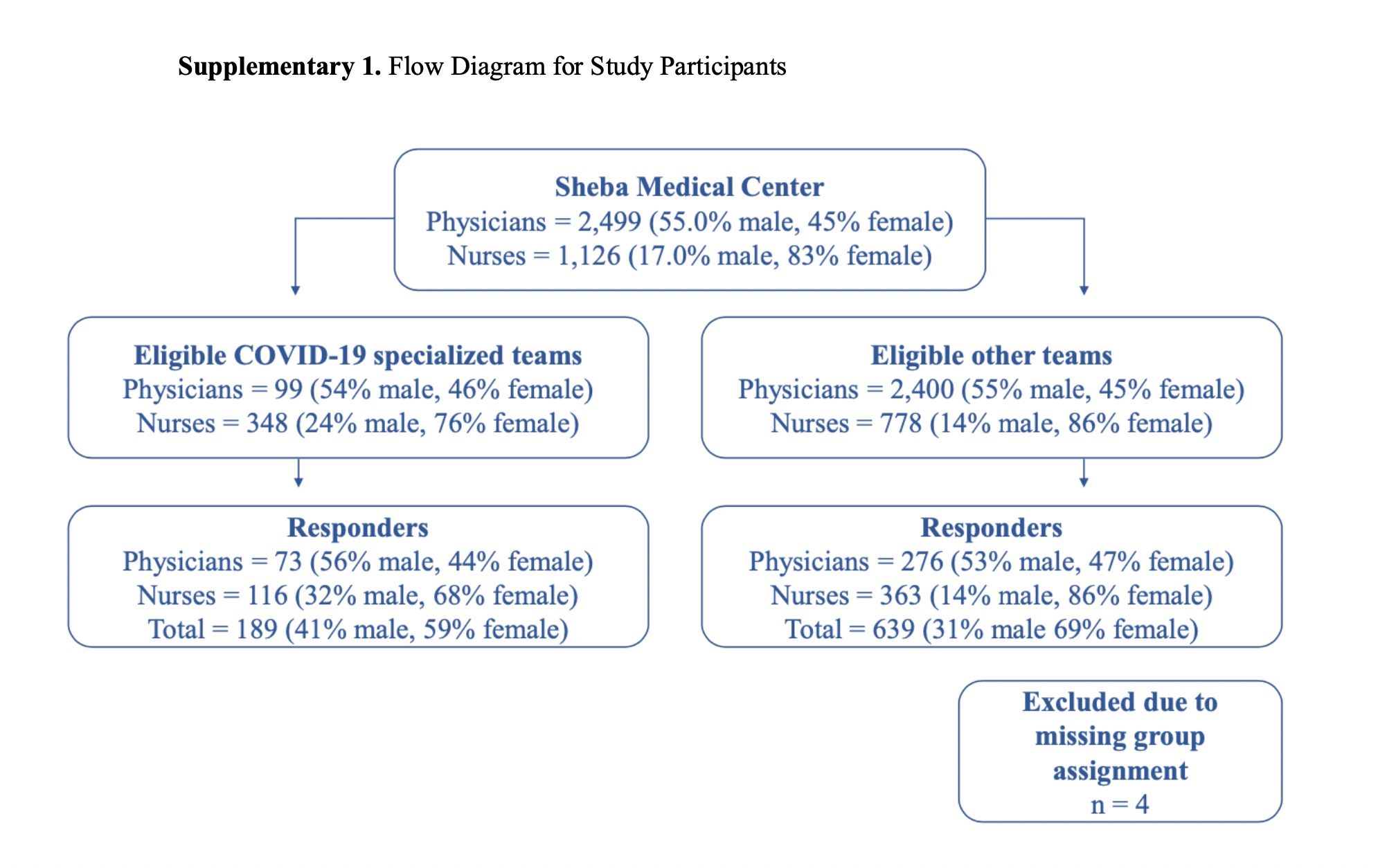

Supplement: Supplementary Figure 1 — Flow diagram for study participants. [file Image_1.JPEG]
